# How to Treat HR+/HER2- Metastatic Breast Cancer Patients after CDK4/6 Inhibitors: An Unfinished Story

**DOI:** 10.3390/life12030378

**Published:** 2022-03-05

**Authors:** Viola Cogliati, Serena Capici, Francesca Fulvia Pepe, Pierluigi di Mauro, Francesca Riva, Federica Cicchiello, Claudia Maggioni, Nicoletta Cordani, Maria Grazia Cerrito, Marina Elena Cazzaniga

**Affiliations:** 1 Phase 1 Research Centre, ASST Monza, 20900 Monza, MB, Italy; capici-400765@asst-monza.it (S.C.); francesca.pepe@asst-monza.it (F.F.P.); marina.cazzaniga@asst-monza.it (M.E.C.); 2 Oncology Unit, ASST Monza, 20900 Monza, MB, Italy; p.dimauro001@unibs.it (P.d.M.); f.riva@asst-monza.it (F.R.); f.cicchiello@asst-monza.it (F.C.); claudia.maggioni@asst-monza.it (C.M.); 3 School of Medicine and Surgery, University of Milano Bicocca, 20900 Monza, MB, Italy; nicoletta.cordani@unimib.it (N.C.); mariagrazia.cerrito@unimib.it (M.G.C.)

**Keywords:** metastatic breast cancer, CDK4/6 inhibitors, therapy resistance, treatment sequencing

## Abstract

CDK4/6 inhibitors in association with endocrine therapy represent the best therapeutic choice for either endocrine-sensitive or resistant hormone-receptor-positive advanced breast cancer patients. On the contrary, the optimal therapeutic strategy after the failure of CDK4/6 inhibitors-based treatment still remains an open question worldwide. In this review, we analyze the most studied mechanisms of resistance to CDK4/6 inhibitors treatment, as well as the most significant results of retrospective and prospective trials in the setting of progression after CDK4/6 inhibitors, to provide the reader a comprehensive overview from both a preclinical and especially a clinical perspective. In our opinion, an approach based on a deeper knowledge of resistance mechanisms to CDK4/6 inhibitors, but also on a careful analysis of what is done in clinical practice, can lead to a better definition of prospective randomized trials, to implement a personalized sequence approach, based on molecular analyses.

## 1. Introduction

Breast cancer (BC) is the most frequent cancer worldwide and also an extremely heterogeneous disease. Hormone-receptor-positive (HR+)/human epidermal growth factor receptor type 2 (HER2)-negative is the most common subtype, representing over 75% of BC. Despite the fact that the majority of these patients have an early-stage malignancy at diagnosis, more than 25% of them will relapse. Median survival for advanced breast cancer (ABC) is currently 40.2 months [1].

Recent data [2] have shown that factors associated with late (>10 years from diagnosis) increased risk of recurrence are HR positivity, large tumor size (>20 mm), and positive lymph nodes at diagnosis; in particular, the risk continues to increase in years 10 to 32. It is therefore estimated that approximately 30% of HR+ patients will develop distant recurrences.

Inhibitors of cyclin-dependent kinases 4 and 6 (CDK4/6i) combined with endocrine therapy (ET) are currently the standard of care for patients with HR+/HER2- metastatic breast cancer (MBC). Overall, data from randomized trials indicate a substantial progression-free survival (PFS) benefit in both primary endocrine resistance (defined as relapse while on the first 2 years of adjuvant ET, or disease progression (PD) within the first 6 months of first-line ET for MBC, while on ET), as well as in secondary endocrine-resistance (defined as a relapse while on adjuvant ET but after the first 2 years, or relapse within 12 months of completing adjuvant ET, or PD after 6 months from ET for ABC start, while on ET) disease. Finally, CDK 4/6i significantly increased overall survival (OS) and maintained or improved quality of life (QoL) [3,4].

However, despite these advances obtained with the combination of ET and CDK4/6i, resistance to this treatment inevitably occurs. Mechanisms of resistance are still poorly understood; therefore, the choice of subsequent therapies is often based on clinical experience rather than a molecular-based approach.

In this review, we first summarize the main results of randomized clinical trials of CDK4/6i in both endocrine-sensitive and endocrine-resistant MBC patients, then we provide an overview of the main mechanisms of resistance to this class of drugs and describe the main results of retrospective and prospective trials focusing on CDK4/6i post-progression treatments.

## 2. CDK4/6i Development 

Dysregulation of the complex network that controls the progression in the cell cycle is a characteristic of the tumor disease. Cyclins and cyclin-dependent kinases (CDK) are responsible for the control of the cell cycle. Among CDKs, CDK4/6 are fundamental for progression in the cell cycle.

Association between CDK4/6 with members of cyclin D controls and promotes entry into the cell cycle and progression across the G1 phase. Cyclin D-CDK4/6 complex is able to phosphorylate the protein of retinoblastoma (Rb), allowing the release of transcriptional factor E2F so far overcoming the phase G1 checkpoint. 

The activation of a cascade of downstream signaling promotes the activity of cyclin E/CDK2 complex, phosphorylation of other target proteins, and progression to S phase, which lead to DNA replication. Regulation of CDK4/6 activity is therefore an important step for the transition from a dormancy state to the entrance in the cell cycle [5].

D-type cyclins, which are linked to CDK4/6, are upregulated and modified replying mitogenic signaling from transductional cascades of RAS/mitogen activated protein kinase (MAPK) and phosphatidylinositol 3-kinase (PI3K)/AKT/Mammalian target of rapamycin (mTOR). Indeed, the importance of CDK4/6i in HR+ BC is explained by cyclin D1, a target of signaling from estrogen receptors and also of several mechanisms of endocrine resistance. Cyclin D1, the partner of CDK4/6, is often overexpressed in HR+/HER2- BC, thus leading to continuous activation of the cyclin D1/CDK4/6 complex [6].

Inhibition of CDK4/6 leads to the complete dephosphorylation of Rb, causing the requisition of transcriptional factor E2F and therefore the stop of the cell cycle progression. The efficacy of CDK4/6i is incremented by the combination with drugs that prevent the estrogen-dependent stimulation of tumor cells. ET causes a depletion of cyclin D1 and therefore a decrease in the construction of complexes with CDK4 and CDK6. This is the basis for the association of CDKi with ET in BC.

Since the first FDA approval of palbociclib in combination with aromatase inhibitors (AI) in first-line treatment [7], several randomized phase III trials have been published, showing the efficacy of CDK4/6i added to an ET, as summarized in the table below (Table 1).

Briefly, PALOMA-1 and PALOMA-2 [8,9] have studied palbociclib + AI vs. placebo, MONALEESA-2 [20] ribociclib vs. placebo and MONARCH-3 [21] abemaciclib vs. placebo in patients not previously treated for MBC. PALOMA-3 for palbociclib [14], MONALEESA-3 for ribociclib [16] and MONARCH-2 for abemaciclib [18] have showed the efficacy of CDK4/6i in association with fulvestrant in MBC patients with an endocrine-resistant disease. Taken together, all these studies have demonstrated a significant improvement in PFS and in some cases also in OS.

These important results, together with the data of MONALEESA-7 [11] in premenopausal MBC patients, led to the approval of these three highly selective inhibitors in association with ET.

Exploratory analyses available from randomized clinical trials suggest that the time from randomization to progression/death on second-line therapy, also known as second progression-free survival (PFS2), may not be compromised by the early use of CDK4/6i in association with ET. Moreover, the time to subsequent chemotherapy (CHT) may be delayed. Recently, Munzone et al. [22] demonstrated that the combination of CDK4/6i plus ET compared with ET alone improved PFS2 and time to subsequent CHT. The delay of CHT is of particular importance, considering patients’ quality of life. It is not known at the moment whether the benefit in PFS2 may postpone the onset of endocrine resistance.

Despite these important results, 20–40% of MBC patients are unresponsive to this kind of treatment, and the remaining ones will develop a resistance. As an example, after 12 months of treatment with ribociclib, 17.8% of the patients had progressed and this percentage increased to 37% after 18 months [20].

In the randomized phase III PEARL study, palbociclib + ET (exemestane or fulvestrant, according to the cohort) was compared to capecitabine in a population of postmenopausal patients very similar to that enrolled in the PALOMA-3 trial. The study failed its primary end-point: palbociclib + ET was not superior to capecitabine in both cohorts of patients, even if palbociclib + ET offered a better quality of life, especially in terms of time to deterioration of global health status [23,24].

## 3. Mechanisms of Resistance to CDK4/6i Inhibitors

Some resistance mechanisms are shared by CDK4/6i and ET, as both treatments act on related pathways. Lots of mechanisms of acquired resistance to ET have already been identified, such as upregulation of ER cofactors (FOXA1 for example), cyclins (particularly D and E), CDK proteins (CDK2 and 6), pathways of mitogenic signaling (PI3K and RAS). Alternatively, or in association, a downregulation of CDK inhibitor proteins has been identified (p16, p21, p27) [25]. Endocrine resistance turns out to be related to genomic and epigenetic mechanisms, such as the activating mutation of ESR1 (the most frequent one, detectable in 40% of patients with metastatic disease) and the hypermethylation of estrogen receptor promoters.

Nevertheless, despite the presence of some kind of resistance, the clinical efficacy of CDK4/6i suggests that in general, endocrine-resistant tumors could maintain a sensibility to CDK4/6 inhibition, particularly when combined with ET. For example, in ctDNA analysis of patients included in PALOMA-3 trial, an improvement in PFS has been retrospectively shown even in those patients with activating mutation of ESR1, a well-known mechanism of ET resistance [26]. Again, also during the development of endocrine resistance, a functioning Rb protein can still be available, making the tumor potentially sensitive to the mechanism of inhibition done by CDK4/6i [27].

Current knowledge of molecular mechanisms of resistance to CDK4/6i is very far from being clarified, and it is essentially based on single agent studies using cell lines as a model. However, several studies have shown that the development of acquired resistance is a multistep process. At the very beginning, cells could undergo adaptive changes that would be able to compromise the response duration, and, after a second time, late-resistance mechanisms could be acquired [5,28]. 

From the analysis of ctDNA of patients in the PALOMA-3 trial, two different types of resistance emerged, one characterizing a rapidly progressive disease, and the other one for a disease that progressed later, suggesting that the mutations involved for each subgroup could be different [29]. Intrinsic resistance occurs quickly after the beginning of treatment, causing the absence of the disease’s response. These tumors already have mutations that allow them to escape the action of the CDK4/6i and proliferate even in the presence of the drug. Many mechanisms of intrinsic resistance have been identified to date, and most of them seem to involve the activation of the cyclin D–CDK4/6–Rb pathway.

Acquired resistance is defined as tumor progression occurring after an initial response to the treatment, or later on during the treatment itself. Mutations included in this process are the result of clonal evolution and several different mechanisms can be involved, such as cyclin D–CDK4/6–Rb activation, activation of different proliferation pathways, modification of the tumor microenvironment and metabolism [30]. Several cell cycle specific and nonspecific mechanisms have been described in this setting and are currently arising as potential resistance biomarkers. Regarding cell cycle-related mechanisms, mutation or loss of Rb, loss of FAT1 [31], gain in CCNE1 [32], changes in the signaling of CDK4/6b and CDK2, increase in CDK6 expression, E2F amplification, and CDK7 overexpression have been described. In the contest of cell-cycle-nonspecific mechanisms, activation of the FGFR pathway or of PI3K/AKT/mTOR pathway, loss of ER or progesterone receptor expression, in addition to immune mechanisms and several others have been identified [33,34]. 

Rb, a tumor suppressor, represents the primary target of CDK4/6i, and loss of Rb function, due to mutations or deletions, emerged in preclinical and clinical studies as a driver of both intrinsic and acquired resistance to CDK4/6i. However, the incidence of Rb mutations in HR+ BCs is low (<5%) prior to the treatment with CDK4/6i and even later on. In the ctDNA analysis of patients enrolled in the PALOMA-3 trial, Rb mutation was identified after treatment with palbociclib and fulvestrant only in 6 out of 127 patients (4.7%) [29]. In NeoPalAna, Rb mutations were identified in 3 BC patients, 2 of whom (7.3% of samples) had a disease responsive to palbociclib [35].

Other attempts to identify predictors of resistance include screening of kinome-wide siRNA, with the aim of identifying kinases up- or downregulated after treatment with ribociclib [36]. These authors showed that PDK1 is able to modify ribociclib sensitivity in ER+ MCF-7 BC cells and that a subsequent treatment with GSK2334470 or dinaciclib, a CDK2 inhibitor, was able to restore the sensitivity of ribociclib-resistant cells to CDK4/6i. 

In preclinical studies, CDK4 and CDK6 overexpression was reported to promote resistance to CDK4/6i. In the clinical studies, the levels of expression of CDK4 were associated with resistance to letrozole alone but not to palbociclib plus letrozole in the PALOMA-2 trial [37]. Other authors [38] identified increased CDK6 expression as a key-player of acquired resistance after palbociclib treatment in ER+ BC cells. In particular, these authors underlined how the increased CDK6 expression observed in resistant cells is dependent on TGF-b pathway suppression via miR-432-5p expression; in particular, they found that levels of miR-432-5p are higher in primary breast cancers, so far demonstrating CDK4/6 resistance compared to those that are sensitive. 

De Leeuw et al. [39] recently showed that an enrichment for enhanced MAPK signaling was present along different resistance models, thus suggesting this feature could allow the growth of more aggressive and more pro-metastatic clones.

Regarding cell cycle nonspecific mechanisms, alteration in the PI3K/AKT/mTOR pathway was largely studied. This pathway is activated in 30–40% of BCs and its alterations have already been recognized as a leading event in the evolution towards endocrine-resistance and more recently to CDK4/6i treatment as well. A recent study by Abreu et al. suggests that the decreasing of cyclin D1 expression caused by a PI3K inhibitor can prevent early adaptations to CDK4/6i [28].

Recently, McCartney et al. [40] studied the modulation of tyrosine-kinase-1 (TK1) levels and activity in palbociclib-sensitive and resistant BC cell lines, as well as in 46 patients enrolled in the TREnd trial. The authors found that TK1 was downregulated after a short-term treatment with palbociclib and that an early reduction of TK1 activity occurred only in sensitive cells; moreover, patients with TK1 levels above the median at the moment of disease progression had worse outcomes on subsequent therapies.

De Angelis et al. [41] studied the molecular features associated with resistance and sensitivity to CDK4/6i: after having generated a palbociclib-resistant cell line from a parental line, they showed through the transcriptomic analysis of these cell lines that there was an association between high IFN signaling and a reduction of sensitivity to CDK4/6i, suggesting that acquired resistance to palbociclib could be associated with activation of the IFN pathway. 

Despite the efforts done to establish predictive factors, no final data at the moment are available.

An important ongoing retrospective analysis, comparing the genomic signature obtained by tissue biopsy of two cohorts of patients, one after treatment with CDK4/6i and the other not, could add useful information. Already available results show that patients post CDK4/6i have a significantly higher tumor mutation burden compared to patients not pretreated with the same drugs (median 2.92 vs. 1.67, *p* < 0.0001), and higher frequency of ESR1 alteration [42].

Having a deeper knowledge of biological resistance mechanisms is therefore really significant for clinicians to improve and customize the best treatment sequence for each patient.

## 4. Treatment Strategies after CDK4/6i: What Is Known, What Is Ongoing, What Is the Future? 

### 4.1. General Strategies: CHT or ET?

The choice of subsequent treatments after CDK4/6i is challenging, because it remains unclear how this class of drugs could modify tumor biology and how already-known therapies could act in such a new context. No guidelines identifying the optimal therapeutic sequence are available.

Pharmacological agents used after CDK4/6i include ET-based regimens (alone, or in combination with other target agents, such as mTOR inhibitors) or CHT. However, few and even sometimes incomplete data are available concerning the real efficacy of the different treatments after CDK4/6i progression. Most of these strategies have been studied before the advent of CDK4/6i, therefore their efficacy in this new context is not yet demonstrated.

Till now, the main data have come from retrospective analyses on a limited number of patients. 

The first data are those from patients enrolled in the phase II PALOMA-1 trial: in that study, the duration of the subsequent standard therapies, ET or CHT, was not significantly different for patients pretreated with palbociclib + letrozole or letrozole alone [43].

In the retrospective analysis led by Xi J et al. [44] conducted among 104 patients who received a next-line treatment after palbociclib, the most frequent choice was CHT, (70%), while 32 patients (30%) received ET. Although a comparison between ET and CHT in terms of efficacy was not possible because of patients’ heterogeneity and selection bias, the authors seemed confident about the efficacy of ET alone after CDK4/6i. Indeed, median PFS with ET alone, or in combination with target agents after first, second, or subsequent lines were 17.0, 9.3, and 4.2 months, respectively. The main limits of this analysis are represented by the small sample size (*n* = 32) and the inclusion of both single agents and combination regimens (everolimus and exemestane, 12 (37.5%)). 

Another retrospective study [45] included 37 patients who developed PD after an initial response to CDK4/6i. Within this group, the most commonly prescribed therapy was a single hormonal agent (29.7%), followed by everolimus/exemestane combination (27%) and single-agent CHT (21.7%). Median time to treatment failure (TTF) was 13.2 months for everolimus/exemestane, 3.1 months for an endocrine single agent, and 4.1 months for CHT single agent. The authors concluded that the results obtained by the combination of everolimus and exemestane in terms of median duration of treatment (>12 months) support the start of further studies to deeper analyze those treatments focused on the PI3K/mTOR pathway after CDK4/6i progression.

Analysis of data concerning patients enrolled in the phase III Paloma-3 trial showed a duration of the treatment immediately following the combination of palbociclib + fulvestrant versus only fulvestrant of respectively 4.9 months vs. 6 months. Median duration of second-line treatment was 5.6 months for CHT and 4.3 months for an everolimus-based combination, supporting the conclusion that previous treatment with palbociclib does not affect the efficacy of the subsequent choice [46].

One of the first available analysis in the setting of systemic therapy beyond progression to CDK4/6i, included 525 postmenopausal MBC patients, identified by an insurance database (MarketScan) in the US. Interestingly, among the subgroup receiving CDK4/6i as first-line treatment, the majority of patients received as subsequent therapy a single endocrine agent (38%); 14.4% the everolimus-based combination, 35.6% CHT, and 9.6% a rechallenge with CDK4/6i. In this analysis, a multivariable regression modeling was employed to evaluate patients’ characteristics: the authors concluded that a first-line treatment combined with fulvestrant (versus AI), rapidly progressive disease and lower age were the main factors associated with the choice for CHT after CDK4/6i [47].

The retrospective analysis of patients enrolled in the phase II TREnd trial analyzed the subsequent treatment of 105 postmenopausal women who previously received palbociclib or palbociclib + ET for moderately pretreated MBC. The median TTF of the treatment received after palbociclib was less than 5 months, with no significant differences according to the prior treatment (palbociclib or ET + palbociclib). Patients who received ET or CHT post-TREnd showed similar mTTF (3.7 months for ET and 4.6 months for CT). In their conclusions, authors speculated that TTF following palbociclib could be independent of prior drug exposure, as their results are similar to those reported in PALOMA-1 and PALOMA-3 trials [48].

A real-world retrospective study, conducted by Basile et al. showed that after a first-line treatment with CDK4/6i, 60% of the patients were treated with CHT and 40% with ET. Patients who received ET had a better prognosis, but the authors failed to identify any significant factor with an influence on the choice of treatment after CDK4/6i [49].

Recently, a retrospective analysis of treatment patterns after progression on palbociclib was conducted in five medical institutions in China. The vast majority of the 200 enrolled patients (73.5%) received CHT, while only 53 patients (26.5%) received ET. No difference was observed between the CHT and ET-treated patients (median PFS 5.5 months) However, in the subgroup of patients resistant to Palbociclib, CHT produced a significant increase in PFS. In contrast, an improvement in PFS with ET was observed only in patients sensitive to prior treatment with palbociclib. The authors concluded that sensitivity to palbociclib could be a predictive factor of response to following treatments [50].

Taken together, these data highlight that ET, as single agents or in combination with other targeted agents, is the preferred choice after CDK4/6i progression, even if an accurate analysis, possibly done on larger studies, could help to clarify the role of CHT in some subgroups of patients. A resume of the principal studies is summarized in Table 2.

### 4.2. Endocrine Agents

AI (letrozole; anastrozole; exemestane); a selective estrogen-receptor modulator (tamoxifen) and a selective estrogen-receptor degrader (fulvestrant) are the most common endocrine agents for the treatment of ABC. One of the potential advantages of fulvestrant versus AI could be represented by its efficacy in tumoral cells harboring ESR1 mutation, that on the other hand can confer resistance to AI [27]. In addition to this, some preclinical data showed that Y537S mutation of ESR1 can be more resistant to fulvestrant compared to D538G mutation [51] and that Y537S can be acquired at disease progression with fulvestrant, as demonstrated both in cellular lines and in ctDNA analysis of patients enrolled in the PALOMA-3 trial. Although the addition of palbociclib to fulvestrant improved PFS regardless of the presence of ESR1 mutation, the emergence of acquired mutation of ESR1 was observed in patients pretreated with palbociclib associated with letrozole or fulvestrant. In the analysis of ctDNA done at baseline and at the end of treatment in PALOMA-3 study, in 25/195 (12.8%) patients a new ESR1 mutation was identified at progression [29]. 

As previously reported, ET is frequently chosen after progression on CDK4/6i, although identifying which patient maintains endocrine sensitivity would be crucial to better direct therapeutic strategy after CDK4/6i. 

In patients previously enrolled in the TREnd trial, a small group of long-responders to subsequent ET was identified, with a mTTF of 13 months, showing a possible significant benefit from continuing ET after a palbociclib-based therapy. However, analysis of these long responders did not show any clinical characteristics associated with such a good response [48].

At the present, different therapeutic strategies targeting the mutation of ESR1, as new SERD or SERM are under development.

Both in the preclinical study [52] and in the phase I studies, the selective estrogen-receptor degrader elacestrant showed significant activity in patients pretreated, also with CDK4/6i [53].

The phase III EMERALD study was designed to evaluate elacestrant as monotherapy versus the investigator’s choice (fulvestrant or AI) for the treatment of HR+/HER2- MBC, previously treated with one to two lines of ET and a maximum one line of CHT, and who had prior progression on ET plus CDK4/6i. A total of 477 patients were enrolled, including 228 with tumors harboring an ESR1 mutation. Elacestrant showed a statistically significant improvement in PFS versus standard ET, with 30% reduction in the risk of progression or death in all patients (HR = 0.697 [95% CI: 0.55, 0.88]; *p* = 0.0018) and 45% reduction in the risk of progression or death in patients with ESR1 mutation (HR = 0.55 [95% CI: 0.39, 0.77]; *p* = 0.0005). The PFS rate at 12 months was 22.32% [54]. Other trials, analyzing elacestrant in association with CDK4/6i or mTOR inhibitors, are already ongoing or planned, in the same subgroup of patients, in earlier lines.

Other important studies are ongoing, to evaluate the efficacy of SERDs, such as the phase I SERENA-1 trial (NCT03616587), designed to evaluate the safety and tolerability of AZD9833 in women with endocrine-resistant HR+/HER2- MBC. The efficacy of AZD9833 as monotherapy or in combination with palbociclib is encouraging, with a dose-dependent safety profile [55]. Results of the phase II study (SERENA-2, NCT04214288), which compares the efficacy and safety of three different doses of AZD9833 versus fulvestrant, are shortly expected.

In addition, the phase II study AMEERA-3 (NCT03616587), is investigating SAR439859 (amcenestrant) versus the physician’s choice in metastatic HR+ BC progressing on >6 months of continuous ET (zero to two lines in the metastatic setting, prior CDK4/6i allowed).

### 4.3. mTOR Inhibitors

The mTOR inhibitor everolimus in association with exemestane was approved for the treatment of MBC HR+/HER2, after the failure of treatment with an AI, on the basis of the results of the phase III BOLERO 2 trial [56], which showed an improvement for PFS for the combination versus exemestane as monotherapy (6.9 versus 2.8 months). The PFS benefit was maintained regardless of any mutations of PIK3CA, FGFR1, or CCND1. In addition to this, in a setting of patients resistant to IA, everolimus leads to better PFS in combination with fulvestrant or tamoxifen. 

However, previous therapy with CDK4/6i was not included in these studies, and at present perspectives studies of exemestane/everolimus combination after CDK4/6i are ongoing.

Some preclinical data suggest significant interaction between the PIK3CA/AKT/mTOR signaling and the downstream CyclinD/CDK4/6/Rb pathway, suggesting that PIK3CA/AKT/mTOR pathway can be implicated in resistance to CDK4/6i [34].

Everolimus in association with ET represents the most popular choice among targeted therapies in clinical retrospective trials.

From the already mentioned analysis of PALOMA-3 data, the median duration of response to everolimus-based treatment was 4.3 months (range 2.5–7.6) when administered after fulvestrant + palbociclib, versus 5 months (2.5–9.4) after single-agent fulvestrant [46]. In the paper by Xi et al., exemestane + everolimus was the preferred choice of ET for 133 patients with progressive disease on palbociclib, and a median PFS of 4.9 months was described [44].

In addition, in a recent retrospective analysis of 41 patients treated with everolimus after progression on palbociclib, a median PFS of 4.2 months was reached, with a median OS of 18.7 months. It has to be underlined that the majority (83%) of patients were treated with three or more prior therapies (median four lines), before palbociclib [57].

Another retrospective cohort study compares efficacy of everolimus + exemestane in patients who progressed on AI alone (26 patients) or AI plus CDK4/6i (17 patients), showing no significant differences in median PFS (respectively 4.2 and 3.6 months) or OS (respectively 11.3 and 15.6 months). Despite all the limitations of really small sample size, this study supports the efficacy of the everolimus/exemestane combination regardless of prior CDK4/6i exposition [58].

At the present, a combination of everolimus with an ET carefully chosen could be therefore considered an effective treatment option after progression on CDK4/6i.

Based on some preclinical evidence showing that the association between mTOR1/2, CDK4/6 and ER inhibition could delay the onset of treatment resistance, some trials are ongoing to evaluate the combination of CDK4/6i plus everolimus and exemestane.

In particular, TRINITI-1 trial, a single arm phase I/II trial, analyzed the combination of ribociclib with exemestane + everolimus in HR+/HER2- MBC, which progressed after prior CDK4/6i. The results showed a clinical benefit rate of 41% at week 24 and the study met its primary endpoint. It was also underlined that in patients with ESR1 or PIK3CA mutations, identified at baseline with ctDNA, a shorter median PFS was identified [59].

### 4.4. PI3K Inhibitors

Activating mutations of the gene PIK3CA have been identified in approximately 35% of HR +/HER2- MBC, and can be acquired during treatment with CDK4/6i.

In the PALOMA-3 trial, at the end of study-treatment, PIK3CA mutations were identified in 52 patients (26.7%), and in 8.2% of patients the variant of PIK3CA was acquired during the treatment [29].

In the phase III trial SOLAR-1 [60], patients with the endocrine-sensitive disease who had progressed to AI were randomized to receive a combination of fulvestrant and alpelisib, a highly selective inhibitor of the PI3K alpha subunit, versus fulvestrant alone. In the subgroup of patients with activating mutations of PI3KCA (exons 7, 9, or 20) median PFS was 11 months (95% CI: 7.5–14.5) for the combination therapy versus 5.7 months (95% CI: 3.7–7.4) for fulvestrant alone. Although only a few patients (*n*.20) in SOLAR-1 trial had received CDK4/6i as previous treatment, median PFS in this subgroup of patients was 5.5 months with the combination versus 1.8 with fulvestrant (HR 0.48, 95% CI: 0.17–1.36). These data suggest efficacy of alpelisib in this setting.

The phase II BYLieve trial was specifically designed to assess the efficacy of alpelisib in combination with ET (fulvestrant or letrozole) after CDK4/6i in MBC HR+/HER2-. At a median follow-up of 11.7 months, among the 121 patients of cohort A (treatment immediately after progression on CDK4/6i and AI) 61 patients (50.4%) were alive without disease progression at 6 months, with a median PFS of 7.3 months [61].

PI3K inhibitors in combination with CDK4/6i have already shown a synergic effect and high efficacy in preclinical studies [62]. Currently, several ongoing clinical trials are investigating triplet combinations of ET, CDK4/6i, and PIK3CA inhibitors.

The pathway of PI3K can be associated with CDK4/6i resistance also through alterations of PTEN (loss) and AKT1 (amplification or mutation). Some trials are already ongoing to evaluate the efficacy of treatments against these specific targets. One of the most intriguing studies is the phase I TAKTIC trial (NCT03959891), which is analyzing the safety and tolerability of ipatasertib (an inhibitor of AKT) in combination with an AI or fulvestrant, with or without palbociclib. The investigators hypothesize that through different combinations of ipatasertib and standard of care drugs, resistance to treatment could be delayed [63].

### 4.5. CHT 

In the case of significant clinical progression and need of rapid symptom relief and disease control, international guidelines suggest using a combination of CHT agents. In the absence of contraindications, anthracyclines or taxane-based regimens are considered the standard of care for patients who didn not receive these therapies in (neo)-adjuvant settings. Other potential options could include capecitabine and vinorelbine, eribulin, gemcitabine, platinum-derived drugs, a different taxane, or liposomal anthracyclines. The choice is always individualized, and should take into account different toxicity profiles, previous exposure to the same drugs and the preferences of the patients. 

In the already-mentioned analysis of Xi J [44], the most frequent choices after palbociclib were capecitabine (21/104, 20.2%), eribulin (16, 15.4%), and nab-paclitaxel (15, 14.4%). It is important to underline that the majority of patients enrolled in the analysis received palbociclib in a setting of endocrine resistance, as a third or subsequent line of treatment.

In the study by Basile et al., among the majority of patients (60%), who received CHT after the first-line treatment with CDK4/6i, 24 patients were treated with capecitabine, 2 with nab-paclitaxel, 2 with vinorelbine, and 1 with paclitaxel. As we could expect, a worse prognosis was identified for patients treated with CHT after CDK4/6i. The authors analyzed the potential factors that could have influenced the choice of second-line treatment, but they didn’t find any that seems to drive the choice of subsequential ET or CHT [49].

In Li et al., analysis of 200 patients, among 147 patients that received CHT after palbociclib, 76 patients received mono CHT. The most frequent single-agent treatments, were: taxane (*n* = 29), capecitabine (*n* = 21), and vinorelbine (*n*= 17). A further 17 patients were treated with anti-vascular endothelial growth factor receptor drugs, and for the other 54 patients the choice was a combination CHT regimen. Also in this case, it is important to remember that the majority (55.5%) of the patients enrolled had received more than two lines of systemic treatment before palbociclib [50].

### 4.6. CDK4/6i beyond Progression

A very important issue for clinical practice is whether the continuation of the same or another CDK4/6i after progression could still be effective.

Preclinical data are discordant, as some studies show that CDK4/6i doesn’t have cross-resistance in cellular models, while other studies highlight some mechanism of resistance shared between different CDK4/6i [64].

In the study by Xi et al., 7 out of 104 patients treated with palbociclib and letrozole were subsequently treated after disease progression with the same CDK4/6i in association with fulvestrant: 3 of these 7 patients received the same CDK4/6i immediately following palbociclib and letrozole and only for these patients a prolonged disease control was shown [44]. 

Similarly, in the real-world study by Princic et al., a small percentage (9.6%) of patients continued with a CDK4/6i in second-line treatment after CDK 4/6 progression [47].

In the multicenter retrospective study by Wander et al., results with abemaciclib, both in association with ET or alone, after progression on palbociclib showed a median PFS of 5.3 months and a median OS of 17.2 months: the authors pointed out the similarity between these results and those reported in MONARCH-1 study. In addition, a subgroup of 32 patients (36.8%) received abemaciclib for more than 6 months. No significant difference was found between patients that received abemaciclib in combination with ET (the majority) or alone [65].

Another single-center retrospective analysis included 30 women who had received palbociclib, in association with AI (77.7%) or fulvestrant (23.3%). After the first disease progression they continued to receive palbociclib, mainly with fulvestrant (56.7%) or AI/tamoxifen, or abemaciclib (2 patients); PFS was 11.8 months. As the majority of the patients received CDK4/6i in association with fulvestrant after progression on palbociclib + AI, the authors concluded that these favorable results could derive also from the efficacy of CDK4/6i + fulvestrant as the first or second line, as shown in previous studies [66].

In addition, a retrospective single institution analysis presented at ASCO 2019, showed that in a subgroup of patients with rapid progression on first treatment with palbociclib or ribociclib, a radiological response was obtained with subsequent treatment with abemaciclib [67].

Different trials are ongoing to further explore this strategy:

(1) TRINITI-1 trial (previously reported preliminary results) is analyzing the addition of ribociclib to everolimus and exemestane in patients pretreated with CDK4/6i [59].
(2) PACE trial (palbociclib after CDKand ET, NCT03147287), phase II randomized trial that aims to compare PFS of fulvestrant alone (arm A) with PFS of fulvestrant + palbociclib (arm B) and of fulvestrant + palbociclib + avelumab (arm C), in patients progressed on treatment with CDK4/6i + ET.
(3) PALMIRA trial, phase II, in which palbociclib is continued with a change in the type of associated ET, in patients who showed prior benefit to the same CDK4/6i.
(4) MAINTAIN study, phase II, is evaluating ribociclib with fulvestrant after treatment with CDK4/6i and AI.

In addition, inhibitors of different CDK are under evaluation. Promising results are awaited from a new CDK2/4/6 inhibitor, PF-06873600, that is being investigated in a phase I trial (NCT03519178).

Recently, the addition of the CDK7 inhibitor samuraciclib to fulvestrant in patients that had previously received CDK4/6i, demonstrated significant activity in a single-arm cohort study, presented at the 2021 San Antonio Breast Cancer Symposium. The authors showed that 72% (18 of 25) patients had tumor shrinkage. In 19 patients (76%) expressing the TP53 wild type and 6 patients expressing TP53 mutant type, the median PFS was 32 weeks and 7.9 weeks, respectively (HR, 0.17; 95% Cl, 0.05–0.53; *p* = 0.0008). They conclude that samuraciclib appears effective, particularly in patients with wild-type TP53 status measured by ctDNA [68].

### 4.7. BCL-2 Inhibitors

BCL-2 is an antiapoptotic protein, overexpressed in 75–85% of HR+ primary BC.

Venetoclax is an oral and selective inhibitor of BCL2 that showed promising results in a phase Ib trial, in association with tamoxifen, in HR+/ BCL2+ MBC, mainly in patients with ESR1 mutation [69]. The phase II VERONICA trial investigated the efficacy of venetoclax associated with fulvestrant, versus fulvestrant alone, in the second/third line of treatment, after progression on CDK4/6i. The primary analysis, presented at ASCO 2021, didn’t show among 103 patients enrolled, any improvement in PFS or clinical benefit rates for the combination, and median PFS did not exceed 3 months for both arms (2.69 for the combination, 1.94 for fulvestrant alone) [70].

### 4.8. Immunotherapy

In addition to the main activity on cell proliferation, CDK4/6i has demonstrated to potentially increase cytotoxic T-cell activity in preclinical data, by upregulating antigen presentation in tumor cells and suppressing the proliferation of regulatory T cells [71]. CDK4/6 inhibition was also shown to be able to block the PDL1 degradation mediated by the proteasome and to enhance PD-1 expressing T cell [72].

The phase 1b JPCE trial was designed to evaluate abemaciclib in combination with pembrolizumab in MBC HR+/HER2- or stage IV nonsmall cell lung cancer. In the breast cohort, 28 patients have been enrolled, obtained an overall response rate of 14.3%, without a correlation with PDL-1 expression, and the treatment showed a manageable safety profile [73].

As reported above, the PACE trial is evaluating the anti PDL-1 avelumab in combination with CDK4/6i, in the specific subset of patients who had progressed to CDK4/6i.

## 5. Conclusions

First-line therapy with CDK4/6i currently represents the gold standard for the initial treatment of MBC HR+/HER2- patients.

The availability of CDK4/6i was one the most revolutionary events in the history of ABC treatment: for the first time, an improvement in OS in such a long natural history cancer population was observed, reaching so far one of the goals of Global Alliance against Cancer.

However, despite the unbelievable benefit of these agents for so many MBC patients, resistance to CDK4/6i still remains a big matter of debate, mainly considering that:

- No prospectively validated predictive factors have been established till now, preventing us to offer a different treatment to potentially unresponsive patients
- No prospective sequential strategies have been developed, so far we still make our own decisions for subsequent therapies in an empirical way

In this review, we analyzed the most important clinical data available till now, to offer a comprehensive overview of the current and ongoing options of treatment after failure of CDK4/6i.

To date, as suggested by clinical guidelines, after a first-line therapy with CDK4/6i and ET, treatment based on endocrine agents still represents an important possibility. Probably, single-agent ET is not enough efficacious in terms of disease control, as shown for the control arm in EMERALD and VERONICA trials; this could be particularly true in patients with bulky disease, or in those with a short response duration of first-line strategy [54,70].

The combination of everolimus and exemestane, a well-known and well-tolerated treatment, could represent one of the main choices, even if the variable results in different retrospective trials needed to be confirmed in a large real-world prospective collection. 

Taking into account the most promising therapeutic options, we are surely waiting for the results of prospective combination trials, that are mainly analyzing (1) the prosecution of the same or a different CDK4/6i, associated with different endocrine backbones, (2) the everolimus combinations, and (3) the role of immunotherapy. In such a heterogeneous disease as HR+/HER2- BC is, more efforts have to be made to find specific biomarkers that can address a personalized treatment approach. Several other mechanisms of resistance are under investigation, and we are looking forward to these results. 

The importance of finding serum biomarkers that can really predict the residual endocrine sensitivity of MBC HR+/HER2- after CDK4/6i, and the potential efficacy of additional target therapy, is also an important and yet unanswered clinical question. In particular, biomarkers that need to be validated in clinical trials are ESR1 mutations, ER expression, and serum thymidine kinase-1 activity (Tka), as well as the alteration in Rb pathway and PIK3CA mutations [74].

The optimal therapeutic strategy after the failure of CDK4/6i-based treatment is still an open question. In this setting, an approach based on a deeper knowledge of resistance mechanisms to CDK4/6i, but also on a careful analysis of what is done in the clinical practice, can lead to the definition of prospective randomized trials, to finally define a personalized sequence of treatment approach.

## Figures and Tables

**Table 1:** Key clinical trial investigating CDK4/6i.

Clinical Trial	Phase	N°	Menopausal Status	Therapeutic Regimen	PFS (Months)	OS (Months)
*1° Line*						
PALOMA-1 TRIO-18 [7,8]	II	165	Post-MP	Palbociclib + letrozole vs. letrozole	20.2 vs. 10.2; HR 0.49 (95% CI: 0.32–0.75); *p* = 0.0004	37.5 vs. 34.5; HR 0.89 (95% CI: 0.62–1.29); *p* = 0.281
PALOMA-2 [9]	III	666	Post-MP	Palbociclib + letrozole vs. letrozole	24.8 vs. 14.5; HR 0.58 (95% CI: 0.46–0.72); *p* < 0.001	Immature data
MONALEESA-2 [4,10]	III	668	Post-MP	Ribociclib + letrozole vs. letrozole	25.3 vs. 16.0; HR 0.57 (95% CI: 0.46–0.70); *p* < 0.0001	63.9 vs. 51.4; HR 0.76 (95% CI: 0.63–0.93); *p* = *0*.004
MONALEESA-7 [11,12]	III	660	Pre-MP	Ribociclib + OFS + tamoxifen/AI vs. OFS + tamoxifen/AI	23.8 vs. 13.0; HR 0.55 (95% CI: 0.44–0.69); *p* < 0.0001	58.7 vs. 48; HR 0.76 (95% CI: 0.61–0.96)
MONARCH-3 [13]	III	493	Post-MP	Abemaciclib + AI vs. AI	28.18 vs. 14.76; HR 0.54 (95% CI: 0.418–0.69); *p* = 0.000002	Immature data
*Postendocrine treatment*						
PALOMA-3 [14,15]	III	521	Post-MP + Pre-MP	Palbociclib + fulvestrant +/− OFS vs. fulvestrant +/− OFS	9.5 vs. 4.6; HR 0.46 (95% CI: 0.36–0.59); *p* < 0.0001	34.8 vs. 28.0; HR 0.81 (95% CI: 0.65–0.99); *p* = 0.022
MONALEESA-3 [16,17]	III	752	Post-Mp	Ribociclib + fulvestrant vs. fulvestrant	20.5 vs. 12.8 HR 0.59 (95% CI: 0.48–0.73); *p* < 0.001	53.7 vs. 41.5; HR 0.73 (95% CI: 0.59–0.90)
MONARCH-2 [18,19]	III	669	Post-MP	Abemaciclib + fulvestrant vs. fulvestrant	16.4 vs. 9.3; HR 0.55 (0.45–0.68); *p* < 0.001	46.7 vs. 37.3 HR 0.75 (95% CI: 0.60–0.94); *p* = 0 .01

**Abbreviations**: N°: number of patients; AI: aromatase inhibitor; OFS: ovarian function suppression; PFS: progression-free survival; OS: overall survival; Post-MP: post-menopausal; Pre-MP: premenopausal.

**Table 2:** Summary of retrospective studies and results of treatment post CDK4/6i.

	Setting	Number of Patients	Treatment after CDK4/6i (All Lines/If Available Second Line)	Efficacy
Xi et al. (2019) [44]	Retrospective single institution analysis of treatment with palbociclib and subsequent regimens	tot: 104 2nd l: 14	All lines: CT: 70 pts - Capecitabine:21 pts - Eribulin:16 pts - Nab-paclitaxel:15pts ET: 32 pts - single agent ET: 16 pts - ET + target therapy: 16 (12 eve + exe)	mPFS: CT: 4.2 m ET: 5.6 m mPFS for second line: not reached for CT ET +/− target agents: 17 m
Giridhar et al. (2018) [45]	Single institution retrospective review of pts who received CDK4/6i treatment as first or second line	tot: 136 2nd l: 37	Second line: mono-ET: 29.7% eve + exe: 27% mono-CT: 21.7%	TTF: mono-ET: 3.1 m Eve + exe: 13.2 m mono-CTt: 4.1 m
Princic et al. (2018) [47]	Population-based observational study, utilizing administrative claims, to describe treatment after prior CDKi-based treatment.	tot: 525 2nd l: 208	Second line: ET: 79 pts (38%) CT: 74 pts (35.6%) everolimus-based: 30 pts (14.4%) CDKi-based: 20 pts (9.6%)	
Rossi et al. (2019) [48]	Retrospective evaluation of prospective collected data from patients enrolled in TREnd trial. Treatment after palbociclib +/− ET for moderately pretreated mBC (up to two lines of ET and/or one line of CT)	tot: 105	All lines: CT:69 pts - Capecitabine +/−VNR+/−CTX: 30 pts - Taxane-based: 21 pts - Anthracycline-based: 9 pts - Others: 9 ET: 33 pts - mono-ET: 27 pts - eve + exe: 6 pts - Other target tp: 3 pts	mTTF: 3.8 m CT: 4.6 m ET: 3.7 m
Basile et al. (2021) [49]	Retrospective multicentric real word study of first- and second-line treatment strategies for 717 pts; including subgroup treated with ET + CDK4/6i in the first line.	2nd l: 48	Second line: CT: 29 pts - Capecitabine: 24 pts - Taxane: 3 pts - VNR: 2 pts ET: 19 pts	CT worse prognosis: HR 6.95, *p*: 0.01
Y Li, W Li et al. (2021) [50]	Retrospective multicentric study of treatment after palbociclib-based treatment (55.5% received more than two lines before palbociclib)	tot: 200	All lines: CT: 147 pts mono-CT: 76 pts - Taxane: 29 pts - Capecitabine: 21 pts - VNR: 17 pts - Others: 9 CT + anti-VEGFR: 17 Combination CT: 54 ET: 53 pts - mono-ET: 7 - chidamide-based regimen: 21 - Everolimus-based combination: 15	mPFS: 5.5 m CT: 5.6 m ET: 4.6 m

Abbreviations: pts: patients; m: months; CT: CHT; ET: endocrine therapy; eve + exe: everolimus + pxemestane; mPFS: median progression-free survival; t: total; 2nd l: second line; VEGFR: vascular endothelial growth factor receptor.
